# Effect of concurrent mitral valve surgery for secondary mitral regurgitation upon mortality after aortic valve replacement or coronary artery bypass surgery

**DOI:** 10.3389/fcvm.2023.1202174

**Published:** 2023-09-29

**Authors:** Shyamal R. Asher, Chin Siang Ong, Raymond J. Malapero, Mahyar Heydarpour, Gregory W. Malzberg, Jasmine T. Shahram, Thy B. Nguyen, Douglas C. Shook, Stanton K. Shernan, Prem Shekar, Tsuyoshi Kaneko, Rodolfo Citro, Jochen D. Muehlschlegel, Simon C. Body

**Affiliations:** ^1^ Department of Anesthesiology, Rhode Island Hospital, Providence, RI, United States; ^2^ Division of Cardiac Surgery, Department of Surgery, Massachusetts General Hospital, Boston, MA, United States; ^3^ Department of Anesthesiology, Perioperative and Pain Medicine, Brigham and Women’s Hospital, Boston, MA, United States; ^4^ Department of Medicine, Brigham and Women’s Hospital, Boston, MA, United States; ^5^ Department of Anesthesia, Critical Care and Pain Medicine, Beth Israel Deaconess Medical Center, Boston, MA, United States; ^6^ Division of Cardiac Surgery, Department of Surgery, Brigham and Women’s Hospital, Boston, MA, United States; ^7^ Cardio-Thoracic-Vascular Department, University Hospital—San Giovanni di Dio e Ruggi d’Aragona, Salerno, Italy; ^8^ Department of Anesthesiology, Boston University School of Medicine, Boston, MA, United States

**Keywords:** mitral valve surgery, aortic valve surgery, coronary artery bypass surgery, mortality, survival, outcomes, mitral regurgitation, guidelines

## Abstract

**Objectives:**

It is uncertain whether concurrent mitral valve repair or replacement for moderate or greater secondary mitral regurgitation at the time of coronary artery bypass graft or aortic valve replacement surgery improves long-term survival.

**Methods:**

Patients undergoing coronary artery bypass graft and/or aortic valve replacement surgery with moderate or greater secondary mitral regurgitation were reviewed. The effect of concurrent mitral valve repair or replacement upon long-term mortality was assessed while accounting for patient and operative characteristics and mitral regurgitation severity.

**Results:**

Of 1,515 patients, 938 underwent coronary artery bypass graft or aortic valve replacement surgery alone and 577 underwent concurrent mitral valve repair or replacement. Concurrent mitral valve repair or replacement did not alter the risk of postoperative mortality for patients with moderate mitral regurgitation (hazard ratio = 0.93; 0.75–1.17) or more-than-moderate mitral regurgitation (hazard ratio = 1.09; 0.74–1.60) in multivariable regression. Patients with more-than-moderate mitral regurgitation undergoing coronary artery bypass graft-only surgery had a survival advantage from concurrent mitral valve repair or replacement in the first two postoperative years (*P* = 0.028) that did not persist beyond that time. Patients who underwent concurrent mitral valve repair or replacement had a higher rate of later mitral valve operation or reoperation over the five subsequent years (1.9% vs. 0.2%; *P* = 0.0014) than those who did not.

**Conclusions:**

These observations suggest that mitral valve repair or replacement for more-than-moderate mitral regurgitation at the time of coronary artery bypass grafting may be reasonable in a suitably selected coronary artery bypass graft population but not for aortic valve replacement, with or without coronary artery bypass grafting. Our findings are supportive of 2021 European guidelines that severe secondary mitral regurgitation “should” or be “reasonabl[y]” intervened upon at the time of coronary artery bypass grafting but do not support 2020 American guidelines for performing mitral valve repair or replacement concurrent with aortic valve replacement, with or without coronary artery bypass grafting.

## Introduction

Secondary mitral regurgitation (MR) results from a progressive increase in left ventricular volume and mitral annular circumference with decreased regional or global myocardial function, commonly due to ischemic heart disease or aortic valve dysfunction (1). The underlying mechanisms of MR from left ventricular dilation are mitral annular enlargement, distortion of the mitral annulus and subvalvular apparatus, and an increase in interpapillary muscle distance (2). MR is commonly observed intraoperatively in patients undergoing aortic valve replacement (AVR) and/or coronary artery bypass grafting (CABG).

Left ventricular remodeling after repair of coronary arterial or aortic valve disease may reduce the severity of secondary MR, thus potentially reducing the need for concurrent mitral valve repair or replacement (MVR/P) (3, 4). However, when moderate or worse MR is observed prior to coronary revascularization or aortic valve surgery, it is presently debated whether MVR/P should be performed at the time of surgery and whether the correction of MR impacts survival (5, 6). The risks of not performing concurrent MVR/P, including persistent secondary MR and the need for cardiac reoperation, loss of potential long-term benefits in survival, and functional status, need to be balanced against the operative and postoperative morbidity and mortality of concomitant MVR/P (3, 5–9). Further, concomitant MVR/P may significantly improve the risk–benefit profile for later transcatheter aortic valve replacement (TAVR) compared to surgical AVR. However, well-conducted trials have failed to demonstrate improved mortality after concomitant MVR/P for moderate MR (7, 8, 10), and thus, the value of concomitant MVR/P for secondary MR during CABG surgery is still debated. Current Guidelines of the American Association for Thoracic Surgery state that “In patients with moderate IMR undergoing CABG, MV repair with an undersized complete rigid annuloplasty ring may be considered” with Class of Recommendation (COR) IIb, Level of Evidence (LOR) B (11). These guidelines differ slightly from those of the American College of Cardiology and American Heart Association (12) and the European Society of Cardiology and the European Association for Cardio-Thoracic Surgery (13).

This study aimed to examine the effect of concomitant MVR/P upon long-term mortality in patients with moderate or greater MR undergoing surgical CABG and/or AVR. We performed a single-center retrospective study to examine the hypothesis that concomitant MVR/P would decrease mortality after AVR and/or CABG while accounting for other risk factors for mortality. Secondary outcome analyses were performed to examine the effect of MVR/P upon subsequent operation on the mitral valve and readmission for heart failure.

## Patients and methods

### Patient selection

**Figure 1:**
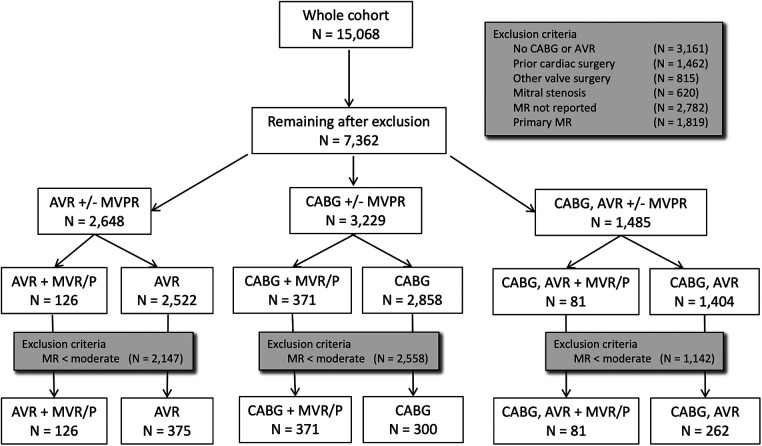
PRISMA diagram. There are two patient exclusion steps. The first step excludes patients not eligible by operative, valvular, or surgical criteria. The second step excludes patients with less-than-moderate severity of mitral regurgitation on both preoperative TTE and intraoperative TEE.

Medical records of 15,068 patients undergoing CABG, AVR, or MVR/P surgery between January 1, 2002, and January 2, 2016, at Brigham and Women's Hospital (BWH) were reviewed. Patients aged <18 or ≥90 years, who did not underwent AVR or CABG surgery, who had mitral stenosis, who underwent prior cardiac surgery, surgery on the tricuspid or pulmonic valve or the ascending aorta, and surgery for endocarditis, who had a missing assessment of MR severity by either preoperative transthoracic echocardiogram (pTTE) or intraoperative transesophageal echocardiogram (iTEE), or with missing mortality information were excluded. Patients with pTTE or iTEE reports describing primary myxomatous disease, chordal rupture, cleft, and perforated, flail, or prolapsing leaflet were also excluded from the analysis. To examine clinically significant MR defined as moderate or greater by either pTTE or iTEE, patients who had mild or less MR by *both* pTTE and iTEE were excluded. This yielded 1,515 patients available for analysis (PRISMA diagram; Figure 1).

### Data collection

Patient demographics and hospital outcomes were obtained from medical records and defined according to the specifications of the Society for Thoracic Surgeons Adult Cardiac Surgery database (14). The primary outcome of all-cause mortality was obtained from institutional follow-up protocols and the U.S. Social Security Death Index. Time to event was calculated from the date of surgery to the date of death or to January 8, 2016. Patients were followed for a median of 8.2 years (IQR 7.93 years). Performance of delayed MVR/P was identified by individual chart review, while subsequent readmission for heart failure was defined as primary admission diagnostic codes (ICD9: 428 and subcodes; ICD10: I05.1, I08.0, I08.1, I08.3, I09.81, I50.1, I50.2, I50.3, I50.4, I50.9, I97.13) occurring after discharge from initial AVR and/or CABG surgery.

### Echocardiographic evaluation

Echocardiographic assessments of the MR grade were recorded from routinely obtained pTTE obtained within 180 days prior to surgery and read by an institutional or community cardiologist. iTEE was obtained intraoperatively prior to cardiopulmonary bypass. Over-reading of images was not performed as the reports constituted the information available to the surgeon. Defined *pre hoc*, we grouped each of the reported pTTE and iTEE patient MR assessments into less-than-moderate, moderate, or more-than-moderate MR. The most severe grade of MR assessed by pTTE or iTEE was used for analysis as it has been previously shown to provide the most predictive value for mortality (15).

### Analysis plan

To test the principal hypothesis that concurrent mitral intervention for moderate or severe MR in patients undergoing CABG and/or AVR substantially improves mortality outcomes, we performed separate analyses on each of the three patient populations—patients who underwent CABG ± MV intervention, patients who underwent CABG and AVR ± MV intervention, and patients who underwent AVR ± MV intervention, while accounting for measured MR severity and other patient and operative characteristics. For each operative cohort, we examined the association with mortality, readmission for heart failure, and reoperation (15).

### Statistical analysis

Statistical analysis was performed using R (https://cran.r-project.org/) and SAS (SAS Institute Inc., Cary, NC, United States) software. Continuous variables are presented as mean (SD) and compared using the unpaired Student’s *t*-test for normally distributed continuous variables. Non-normally distributed variables are described as median and 10–90th percentiles of range and compared using a Kruskal–Wallis test. Categorical variables were compared using the Fisher exact test. Missing data were excluded from the analysis.

Predictors of mortality outcome were described by Kaplan–Meier estimates and analyzed by Cox proportional hazards regression. For these analyses, we selected variables based on their clinical significance, variation between the study cohorts, and known contributions to life expectancy. These included age, gender, chronic obstructive pulmonary disease (COPD), preoperative dialysis, peripheral vascular disease, cerebrovascular disease, history of myocardial infarction, heart failure, preoperative atrial fibrillation, and an LVEF <40%. For exposure and each potential confounder, we first performed univariate analyses of mortality, followed by a stepwise multivariable analysis of potential confounders with a Cox proportional hazards univariate *P*-value <0.20. The year of operation, patient gender, the primary surgical operation, and concurrent mitral valve surgery were forced into the multivariate model. Model overfitting was tested with Cox–Snell residuals. Results are presented as hazard ratios (HRs) with 95% confidence intervals (CIs).

## Results

### Cohort characteristics

Across the three classes of surgery, 1,515 patients with clinically significant MR, graded as moderate or greater by either pTTE or iTEE, were followed over the median 8.2-year follow-up period (Table 1). Overall, patients were more frequently older than 60 years (85%), male (57%), and Caucasian (95%). There were significant differences in cardiac risk factors between operative cohorts (Supplementary Table S3). Comparison of MR severity assessed by pTTE and iTEE showed a preponderance of iTEE assessment of MR severity being less than pTTE severity (Supplementary Table S2). A total of 938 patients underwent AVR and/or CABG surgery alone, while 577 patients underwent concurrent MVR/P. Patients who underwent concurrent MVR/P were younger, more likely to be male, with coronary artery disease, with reduced ejection fraction, without aortic valve disease, and with more severe MR when assessed by either pTTE or iTEE (Table 1). Patients undergoing CABG surgery were more likely to have concurrent MVR/P. Few patients underwent subsequent mitral valve operation or reoperation (0.85%) after the initial surgery.

**Table 1:** Characteristics of the study cohort of 1,515 patients stratified by whether or not a concurrent MVR/P operation was performed.

		AVR and/or CABG without concurrent MVR/P	AVR and/or CABG with concurrent MVR/P	*P*-value
		(*N* = 938)	(*N* = 577)	
Demographics				
Age (years; *N*/%)	<50	29 (3%)	36 (6%)	<0.0001
	50–59	79 (8%)	85 (15%)	
	60–69	161 (17%)	147 (25%)	
	70–79	339 (36%)	208 (36%)	
	≥80	330 (35%)	101 (18%)	
Gender (female; *N*/%)		421 (45%)	224 (39%)	0.024
Race (Caucasian; *N*/%)		887 (95%)	542 (95%)	0.92
BMI strata (kg/m^2^; Caucasian/%)	<20	34 (4%)	28 (5%)	0.25
	20–24.9	254 (27%)	162 (28%)	
	25–29.9	370 (39%)	199 (34%)	
	30–34.9	178 (19%)	113 (20%)	
	35–39.9	71 (8%)	46 (8%)	
	≥40	31 (3%)	29 (5%)	
Co-existing disease				
Current or past smoker (yes; *N*/%)		417 (44%)	285 (49%)	0.069
COPD (yes; *N*/%)		166 (18%)	109 (19%)	0.61
Diabetes (*N*/%)	NIDDM	91 (10%)	64 (11%)	0.52
	IDDM	188 (20%)	105 (18%)	
Dyslipidemia (yes; *N*/%)		737 (79%)	411 (71%)	0.001
Hypertension (yes; *N*/%)		736 (78%)	426 (74%)	0.044
Preop dialysis (yes; *N*/%)		24 (3%)	18 (3%)	0.63
Peripheral vascular disease (yes; *N*/%)		151 (16%)	93 (16%)	1
Cerebrovascular disease (yes; *N*/%)		154 (16%)	86 (15%)	0.48
Medications				
ASA (yes; *N*/%)		602 (64%)	349 (60%)	0.165
Beta-blocker (yes; *N*/%)		505 (54%)	324 (56%)	0.41
ACEI/ARB (yes; *N*/%)		74 (8%)	64 (11%)	0.044
Cardiac disease				
Myocardial infarction (*N*/%)	Past MI	154 (16%)	124 (21%)	0.04
	Recent MI	139 (15%)	86 (15%)	
NYHA class (*N*/%)	I & II	503 (54%)	281 (49%)	0.043
	III & IV	435 (46%)	296 (51%)	
Heart failure (yes; *N*/%)		524 (56%)	343 (59%)	0.19
Preoperative atrial fibrillation (yes; N/%)		53 (6%)	47 (8%)	0.073
Diseased coronary vessels (*N*/%)	None	303 (32%)	109 (19%)	<0.0001
	One	133 (14%)	85 (15%)	
	Two	181 (19%)	128 (22%)	
	Three or more	321 (34%)	255 (44%)	
TEE aortic stenosis (*N*/%)	Less than moderate	380 (42%)	439 (79%)	<0.0001
	Moderate	53 (6%)	23 (4%)	
	More than moderate	480 (53%)	94 (17%)	
TEE aortic insufficiency (*N*/%)	Less than moderate	634 (69%)	440 (77%)	0.001
	Moderate	178 (19%)	71 (12%)	
	More than moderate	102 (11%)	60 (11%)	
Preoperative TTE mitral regurgitation (*N*/%)	Less than moderate	218 (23%)	128 (22%)	<0.0001
	Moderate	681 (73%)	308 (53%)	
	More than moderate	39 (4%)	141 (24%)	
Intraoperative TEE mitral regurgitation (*N*/%)	Less than moderate	511 (54%)	66 (11%)	<0.0001
	Moderate	372 (40%)	293 (51%)	
	More than moderate	55 (6%)	218 (38%)	
Worst grade of mitral regurgitation (*N*/%)	Moderate	853 (91%)	259 (45%)	<0.0001
	More than moderate	85 (9%)	318 (55%)	
LV ejection fraction <40% (*N*/%)		241 (27%)	220 (39%)	<0.0001
Cardiac injury score (*N*/%)	0	365 (39%)	176 (31%)	<0.0001
	1	328 (35%)	199 (34%)	
	2	228 (25%)	188 (33%)	
	3	5 (1%)	10 (2%)	
Operation				
Year of operation (*N*/%)	2002–2003	142 (15%)	147 (25%)	<0.0001
	2004–2005	126 (13%)	98 (17%)	
	2006–2007	134 (14%)	85 (15%)	
	2008–2009	156 (17%)	76 (13%)	
	2010–2011	147 (16%)	85 (15%)	
	2012–2013	176 (19%)	61 (11%)	
	2014–2015	57 (6%)	25 (4%)	
Urgency (Not elective; *N*/%)		352 (38%)	275 (48%)	0.0001
CPB time [min; median (10%–90% CI)]		115 (70–201)	173 (115–266)	<0.0001
Cross clamp time [min; median (10%–90% CI)]		84 (52–147)	129 (84–204)	<0.0001
CABG and or AVR performed (*N*/%)	AVR	375 (40%)	126 (22%)	<0.0001
	CABG	300 (32%)	371 (64%)	
	CABG and AVR	263 (28%)	80 (14%)	
Mitral valve repair or replacement (*N*/%)	Repair	–	445 (77%)	
	Replacement	–	132 (23%)	
Outcomes				
Duration of follow-up (years)		8.1 (7.51)	8.3 (8.48)	0.25
Follow-up index (%)		0.80 (0.04–1.0)	0.70 (0.03–1.0)	0.34
Mortality (yes; N/%)	0–30 days	50 (5.3%)	25 (4.3%)	0.45
	31–365 days	67 (7.5%)	45 (8.2%)	0.75
	1–5 years	151 (18.4%)	103 (20.3%)	0.43
	>5 years	139 (29.7%)	102 (32.0%)	0.37
MVR/P during 5-years of follow-up (yes; *N*/%)		2 (0.2%)	11 (1.9%)	0.0014
Readmission for heart failure (yes; N/%)	0–30 days	20 (2.1%)	23 (4.0%)	0.051
	31–365 days	26 (2.9%)	35 (6.3%)	0.0028
	1–5 years	40 (4.9%)	32 (6.3%)	0.32
	>5 years	20 (4.3%)	22 (6.9%)	0.15

Data are reported as *N* (%), mean (standard deviation), or median (10–90th percentiles).

BMI, body mass index; ACEI/ARB, angiotensin-converting enzyme inhibitor/angiotensin receptor blocker; NYHA, New York Heart Association; TTE, transthoracic echocardiography; CPB, cardiopulmonary bypass; STS, Society of Thoracic Surgeons.

Cardiac injury score was modified from *Eur Heart J*. (2017) 38(45):3351–8.

### Univariate predictors of mortality

**Figure 2:**
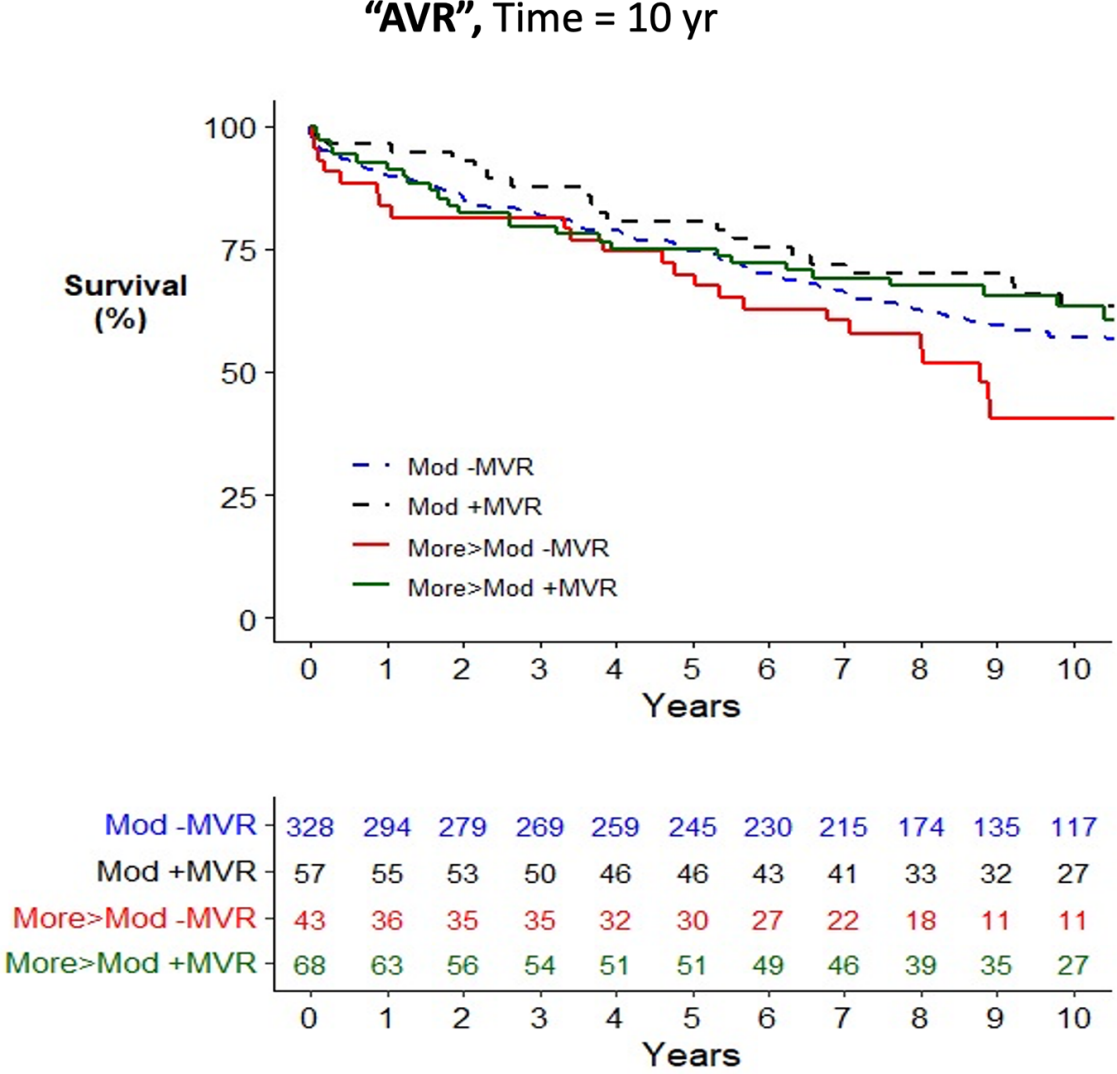
Kaplan–Meier plot of survival of 501 patients undergoing AVR who underwent MVR/P or not, stratified by the worst measured severity of MR. Observed mortality is stratified by the source of the most severe grade of MR (moderate, >moderate) and whether or not MVR/P was concurrently performed. Pairwise comparison of survival between AVR patients with moderate MR who underwent MVR/P or did not showed no statistical significance (*P* = 0.31) after adjustment for two comparisons. Pairwise comparison of survival between patients with more-than-moderate MR who underwent MVR/P or did not showed no statistical significance (*P* = 0.063) after adjustment for two comparisons.

**Figure 3:**
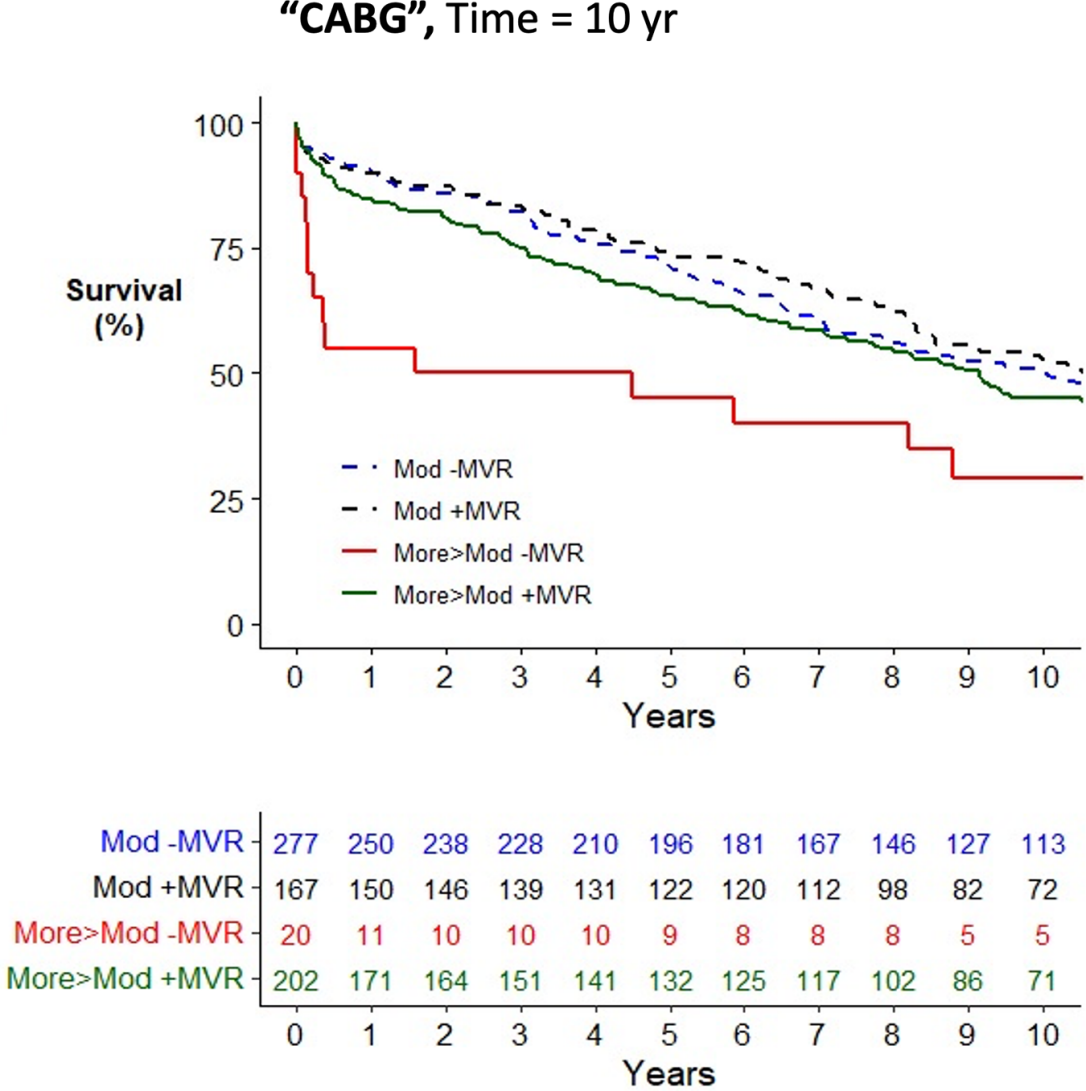
Kaplan–Meier plot of survival of 672 patients undergoing CABG who underwent MVR/P or not stratified by the worst measured severity of MR. Observed mortality is stratified by the source of the most severe grade of MR (moderate, >moderate) and whether or not MVR/P was concurrently performed. Pairwise comparison of survival between CABG patients with moderate MR who underwent MVR/P or did not showed no statistical significance (*P* = 0.49) after adjustment for two comparisons. Pairwise comparison of survival between patients with more-than-moderate MR who underwent MVR/P or did not showed statistical significance when adjusted for two comparisons (*P* = 0.028), which was explained by mortality in the first 2 postoperative years.

**Figure 4:**
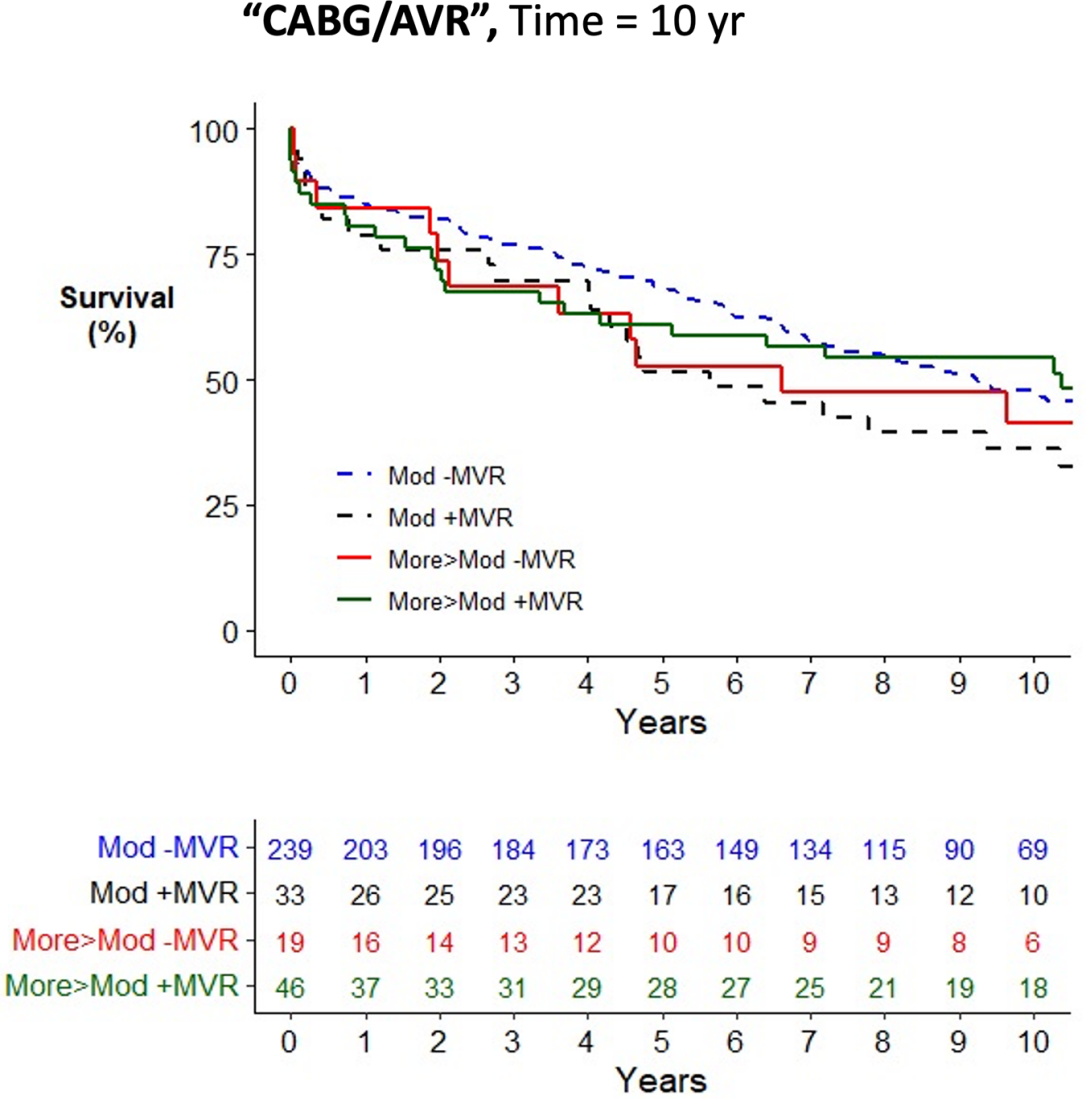
Kaplan–Meier plot of survival of 345 patients undergoing AVR/CABG who underwent MVR/P or not stratified by the worst severity of MR. Observed mortality is stratified by the source of the most severe grade of MR (moderate, >moderate) and whether or not MVR/P was concurrently performed. Pairwise comparison of survival between AVR/CABG patients with moderate MR who underwent MVR/P or did not showed no statistical significance (*P* = 0.15) after adjustment for two comparisons. Pairwise comparison of survival between patients with more-than-moderate MR who underwent MVR/P or did not showed no statistical significance (*P* = 0.55) after adjustment for two comparisons.

Across the cohort, there was an increased risk of mortality associated with older age, female gender, medical comorbidities, and urgent surgery when assessed using a univariate Cox proportional hazards mortality model (Supplementary Table S4). Patients having CABG surgery alone (HR = 1.23; 1.02–1.47) were at increased risk of mortality compared to AVR (HR = 1.00), while patients having combined AVR/CABG were at further increased risk (HR = 1.47; 1.19–1.82). Concurrent mitral valve surgery did not alter the risk of postoperative mortality in the study cohort (HR = 1.00; 0.86–1.17) nor when stratified by moderate MR (HR = 0.94; 0.76–1.15) or severe MR (HR = 0.81; 0.57–1.13). In all operative cohorts, patients with moderate MR, defined as the worst observed severity of MR, had no improvement in survival from concurrent performance of MVR/P (see Figures 2–4 for AVR, CABG, and AVR/CABG, respectively). In patients undergoing CABG-only surgery with the worst MR severity by either pTTE or iTEE being more-than-moderate MR, there was a survival advantage from concurrent MVR/P in the first two postoperative years (*P* = 0.028; Figure 3) but not beyond that period. This was not observed in patients undergoing AVR or AVR/CABG surgery.

### Mortality risk from concurrent MVR/P

To determine whether concurrent MVR/P at the time of CABG and/or AVR improved long-term survival, we used multivariate Cox proportional hazard modeling, stratified by the most severe grade of MR severity observed by either pTTE or iTEE (Supplementary Table S3). Hazards models for each operative class (AVR, CABG, and AVR/CABG; Supplementary Table S5) did not show a survival advantage from concurrent MVR/P after adjustment for other covariates. For patients with moderate MR, there was no observed survival advantage (HR = 0.93; 0.75–1.17) after accounting for other predictors of mortality. Similar findings were observed for patients with more-than-moderate MR (HR = 1.09; 0.74–1.60). There was no evidence of model overfitting (Supplementary Table S6 and Figure S1).

### Readmission for heart failure and mitral valve reoperation

To determine whether concurrent MVR/P at the time of CABG and/or AVR reduced the rate of readmission for heart failure or subsequent MVR/P during the following 5 years, we compared these outcomes by whether concurrent MVR/P was performed (Table 1). Patients who underwent concurrent MVR/P had a higher rate of later mitral valve operation or reoperation over the five subsequent years (1.9% vs. 0.2%; *P* = 0.0014) than those who did not. Similarly, patients who underwent concurrent MVR/P had a higher incidence of readmission with a primary diagnosis of heart failure over the first postoperative year (10.3% vs. 5.0%; Table 1).

## Discussion

In this retrospective study, we compared patients with moderate or greater secondary MR when undergoing CABG and/or AVR and assessed whether concurrent MVR/P improved survival and other outcomes. We observed improved survival in the first 2 years after surgery in a subgroup of patients undergoing CABG surgery with concomitant MVR/P who had more-than-moderate MR. This improvement in survival was not observed in patients undergoing AVR or CABG/AVR surgery nor in patients with moderate MR undergoing any operation. The low frequency of subsequent mitral surgery or admission for heart failure was not significantly reduced by concurrent mitral valve surgery.

Well-conducted randomized trials for the treatment of moderate or severe MR by MVR/P have been performed (10, 16), yielding consensus on the value of MVR/P alone in secondary MR (11–13). These studies have not demonstrated significant improvement in short to intermediate-term mortality after MVR/P but have yielded important insights into the value of mitral valve replacement vs. repair. In contrast, the potential mortality benefit of concurrent MVR/P during primary surgery for CABG and/or AVR has not been demonstrated. This determination is important because expected or unexpected findings of moderate or greater MR during CABG and/or AVR occur intraoperatively, and surgical guidance is lacking.

The benefit of MVR/P concomitant with CABG for moderate MR has been examined in randomized trials (7, 8, 10), several observational studies (17–19), and meta-analyses (22–22). Consensus indicates no survival benefit from concomitant MVR/P with CABG, as we also observed, but studies of survival after concomitant AVR or AVR/CABG are few (23) and limited in scope. Although there is evidence that concomitant MVR/P decreases echocardiographic measures of MR after both AVR and AVR/CABG, survival comparison is generally lacking, but when present, it indicates no survival benefit from concomitant MVR/P (24, 25).

Our results did not reveal a significant survival advantage of performing concomitant MVR/P for either moderate or more-than-moderate MR at the time of AVR or AVR/CABG surgery but may have been limited by the small number of patients with unrepaired severe MR. Our findings are not yet supportive of current European guidelines that severe secondary MR “should” be intervened upon at the time of CABG and/or AVR (Class 1C) (13). Current American guidelines call an intervention in this scenario “reasonable” (Class 2A) (12). Our observations suggest that MVR/P for more than moderate MR at the time of CABG may be reasonable in a suitably selected CABG population but not for AVR with or without CABG. The decision to surgically treat secondary MR has depended on severity, patient comorbidity, and technical complexity. The addition of a second valve surgery to an AVR has been previously shown to increase operative risk (26), which we did not observe. We interpret these findings as not yet justifying performing concurrent MVR/P for moderate or more severe MR in all situations. Unrepaired, moderate, or severe MR has been variably reported to be an independent risk factor for mortality after surgical AVR (SAVR) (6, 27) and after TAVR (27, 28). TAVR allows individual assessment of the reduction of MR severity after treatment of aortic stenosis while not adding the risk of concurrent MVR/P in all patients. Based on the findings of this study and TAVR's ability to provide a window into an assessment of improvement in MR, the role of SAVR and concurrent MVR/P seems weak. The absence of large-scale studies examining the effect of MVR/P concurrent with AVR/CABG upon mortality and our observed lack of benefit seems to also not support its use.

### Limitations

The single-center, retrospective design of this study had inherent limitations, but it allowed for the assessment of long-term postoperative outcomes. Patients were not randomly assigned to undergo concomitant MVR/P; therefore, there is a potential for bias by clinical presentation or surgical practices that are unaccounted for in this study. We could not differentiate between outcomes of mitral valve repair vs. mitral valve replacement nor the severity of MR in follow-up. The statistical techniques used accounted for a number of variables but are unable to account for unmeasured confounders, which can occur in retrospective studies. We used all-cause mortality rather than cardiac-specific mortality as our primary outcome. While this would include mortality not due to primary disease, it allows for a more complete accounting of mortality. We are unable to identify specific indications for reoperations and/or readmissions, as well as those that may have occurred at other institutions, as a cause of mortality. We are also unable to comment on postoperative medical management or postoperative severity of MR.

## Conclusions

In this retrospective study comparing survival in patients with secondary MR undergoing CABG and/or AVR, we assessed whether concurrent MVR/P improved survival. Improved survival was only observed in a small cohort of patients with more-than-moderate MR undergoing CABG surgery and only in the first 2 years after surgery. This improvement in survival was not observed in patients undergoing AVR or CABG/AVR surgery nor in patients with moderate MR undergoing any operation. Our findings suggest that MVR/P for more-than-moderate MR at the time of CABG is reasonable in a suitably selected population but is not indicated when undergoing AVR, with or without CABG.

## Data Availability

The original contributions presented in the study are included in the article/Supplementary Material, further inquiries can be directed to the corresponding author.
