# Supplementary material for: Effect of concurrent mitral valve surgery for secondary mitral regurgitation upon mortality after aortic valve replacement or coronary artery bypass surgery
Source: Front Cardiovasc Med. 2023 Sep 29;10:1202174. doi: 10.3389/fcvm.2023.1202174 (PMC10570832; doi:10.3389/fcvm.2023.1202174)

**Supplementary Table 1: Multivariate Cox proportional hazards model of mortality in each surgical cohort (N=1,515).**

|  | **Spacer** |  | **Multivariate Cox PH for  AVR   (N=501)** | | |  | **Multivariate Cox PH for CABG  (N=671)** | | |  | **Multivariate Cox PH for  AVR/CABG  (N=343)** | | |
| --- | --- | --- | --- | --- | --- | --- | --- | --- | --- | --- | --- | --- | --- |
| **Spacer** |  | **Spacer** | **Hazard ratio** | **Spacer** | **P value** | **Spacer** | **Hazard ratio** | **Spacer** | **P value** | **Spacer** | **Hazard ratio** | **Spacer** | **P value** |
| **Demographics** |  |  |  |  |  |  |  |  |  |  |  |  |  |
| Age (years) | <50 |  | ref |  | - |  | ref |  | - |  | ref |  | - |
|  | 50-59 |  | 1.50 (0.55 - 4.12) |  | 0.43 |  | 2.16 (0.65 - 7.22) |  | 0.21 |  | 1.62 (0.29 - 8.99) |  | 0.58 |
|  | 60-69 |  | 3.22 (1.33 - 7.82) |  | 0.010 |  | 3.48 (1.09 - 11.01) |  | 0.035 |  | 2.58 (0.52 - 12.8) |  | 0.25 |
|  | 70-79 |  | 4.18 (1.75 - 9.98) |  | 0.001 |  | 5.30 (1.68 - 16.69) |  | 0.0044 |  | 4.72 (0.98 - 22.6) |  | 0.052 |
|  | ≥80 |  | 8.87 (3.73 - 21.1) |  | <0.0001 |  | 9.63 (3.03 - 30.7) |  | 0.0001 |  | 5.90 (1.21 - 28.8) |  | 0.028 |
| Gender (Female) |  |  | 0.80 (0.58 - 1.11) |  | 0.18 |  | 1.11 (0.89 - 1.39) |  | 0.34 |  | 1.04 (0.74 - 1.45) |  | 0.83 |
|  |  |  |  |  |  |  |  |  |  |  |  |  |  |
| **Co-existing disease** |  |  |  |  |  |  |  |  |  |  |  |  |  |
| COPD (Yes) |  |  | 2.22 (1.57 - 3.15) |  | <0.0001 |  | 1.69 (1.28 - 2.23) |  | 0.0002 |  | - |  | - |
| Diabetes | NIDDM |  | 2.01 (1.25 - 3.24) |  | 0.0043 |  | 1.22 (0.95 - 1.58) |  | 0.12 |  | 1.13 (0.75 - 1.72) |  | 0.56 |
|  | IDDM |  | 2.37 (1.25 - 4.51) |  | 0.0084 |  | 1.33 (0.97 - 1.82) |  | 0.079 |  | 1.45 (0.83 - 2.54) |  | 0.19 |
| Hypercholesterolemia (Yes) |  |  | 0.66 (0.48 - 0.92) |  | 0.015 |  | - |  | - |  | - |  | - |
| Preoperative dialysis (Yes) |  |  | 6.97 (3.11 - 15.6) |  | <0.0001 |  | 3.72 (2.17 - 6.40) |  | <0.0001 |  | 5.91 (3.01 - 11.6) |  | <0.0001 |
|  |  |  |  |  |  |  |  |  |  |  |  |  |  |
| **Cardiac disease** |  |  |  |  |  |  |  |  |  |  |  |  |  |
| Heart failure (Yes) |  |  | - |  | - |  | - |  | - |  | 1.60 (1.14 - 2.25) |  | 0.007 |
| Preoperative atrial fibrillation (Yes) |  |  | - |  | - |  | - |  | - |  | 1.57 (0.94 - 2.60) |  | 0.084 |
| Worst grade of mitral regurgitation (More than moderate *) |  |  | 1.22 (0.81 - 1.83) |  | 0.34 |  | 1.27 (0.98 - 1.65) |  | 0.069 |  | 1.20 (0.76 - 1.89) |  | 0.43 |
| Number of diseased vessels | Two |  | - |  | - |  | 2.37 (1.28 - 4.36) |  | 0.0058 |  | - |  | - |
|  | Three or more |  | - |  | - |  | 2.19 (1.20 - 4.00) |  | 0.010 |  | - |  | - |
|  |  |  |  |  |  |  |  |  |  |  |  |  |  |
| **Operation** |  |  |  |  |  |  |  |  |  |  |  |  |  |
| Urgency of operation (Not elective) |  |  | 1.47 (1.03 - 2.08) |  | 0.033 |  | 1.21 (0.96 - 1.54) |  | 0.11 |  | - |  | - |
| Mitral valve operation (Yes) |  |  | 1.02 (0.68 - 1.53) |  | 0.92 |  | 1.02 (0.78 - 1.31) |  | 0.90 |  | 1.09 (0.72 - 1.65) |  | 0.67 |
|  |  |  |  |  |  |  |  |  |  |  |  |  |  |
|  | R2 |  | 0.207 |  |  |  | 0.198 |  |  |  | 0.165 |  |  |
|  | Concordance |  | 0.719 |  |  |  | 0.676 |  |  |  | 0.674 |  |  |
|  | LRT |  | 115.8 |  |  |  | 139 |  |  |  | 61.65 |  |  |
|  | LRT df |  | 19 |  |  |  | 20 |  |  |  | 19 |  |  |
|  | LRT P value |  | <0.0001 |  |  |  | <0.0001 |  |  |  | <0.0001 |  |  |
|  |  |  |  |  |  |  |  |  |  |  |  |  |  |
| *  Reference group is patients with moderate MR | |  |  |  |  |  |  |  |  |  |  |  |  |

**Supplementary Table 2: Univariate Cox proportional hazards model of survival for 1,515 patients undergoing AVR and/or CABG, with or without concurrent MVR/P.** Results are portrayed as hazard ratio and 95% confidence interval of the hazard ratio. AVR, aortic valve replacement; CABG, coronary artery bypass graft; BMI, body mass index; COPD, chronic obstructive pulmonary disease; ACEI/ARB, angiotensin converting enzyme inhibitor / angiotensin receptor blocker; NYHA, New York Heart Association; TTE, transthoracic echocardiography; pTTE, preoperative TTE; TEE, transesophageal echocardiography; iTEE, intraoperative TEE; CPB, cardiopulmonary bypass; STS, Society of Thoracic Surgeons; MVR/P, mitral valve repair or replacement.

| **Spacer** |  | **Spacer** | **Hazard ratio** | **Spacer** | **P value (level)** | **Spacer** | **P value (overall)** |
| --- | --- | --- | --- | --- | --- | --- | --- |
| **Demographics** |  |  |  |  |  |  |  |
| Age (years) | <50 |  | ref |  | - |  | <0.0001 |
|  | 50-59 |  | 1.71(0.88 - 3.32) |  | 0.11 |  |  |
|  | 60-69 |  | 2.73(1.47 - 5.06) |  | 0.0015 |  |  |
|  | 70-79 |  | 4.15(2.27 - 7.58) |  | <0.0001 |  |  |
|  | ≥80 |  | 6.00(3.27 - 11.0) |  | <0.0001 |  |  |
| Gender | Female |  | 1.23(1.06 - 1.45) |  | 0.005 |  |  |
| Race | Caucasian |  | 1.37(0.93 - 2.00) |  | 0.11 |  |  |
| BMI strata(kg/m2) | <20 |  | 1.56(1.09 - 2.22) |  | 0.015 |  | 0.26 |
|  | 20-24.9 |  | 1.14(0.95 - 1.38) |  | 0.16 |  |  |
|  | 25-29.9 |  | ref |  |  |  |  |
|  | 30-34.9 |  | 1.05(0.85 - 1.30) |  | 0.67 |  |  |
|  | 35-39.9 |  | 1.06(0.79 - 1.43) |  | 0.70 |  |  |
|  | ≥40 |  | 0.99(0.64 - 1.51) |  | 0.95 |  |  |
|  |  |  |  |  |  |  |  |
| **Co-existing disease** |  |  |  |  |  |  |  |
| Smoker |  |  | 1.12(0.97 - 1.31) |  | 0.12 |  |  |
| COPD |  |  | 1.62(1.35 - 1.94) |  | <0.0001 |  |  |
| Diabetes | NIDDM |  | 1.52(1.21 - 1.91) |  | 0.0003 |  |  |
|  | IDDM |  | 1.32(1.10 - 1.59) |  | 0.031 |  |  |
| Dyslipidemia |  |  | 1.02(0.86 - 1.22) |  | 0.79 |  |  |
| Hypertension |  |  | 1.63(1.34 - 1.97) |  | <0.0001 |  |  |
| Preoperative dialysis |  |  | 4.37(3.11 - 6.16) |  | <0.0001 |  |  |
| Peripheral vascular disease |  |  | 1.55(1.29 - 1.85) |  | <0.0001 |  |  |
| Cerebrovascular disease |  |  | 1.44(1.19 - 1.75) |  | 0.0002 |  |  |
|  |  |  |  |  |  |  |  |
| **Medications** |  |  |  |  |  |  |  |
| ASA |  |  | 0.95(0.82 - 1.11) |  | 0.54 |  |  |
| Beta blocker |  |  | 1.04(0.89 - 1.21) |  | 0.60 |  |  |
| ACEI/ARB |  |  | 1.08(0.85 - 1.36) |  | 0.54 |  |  |
|  |  |  |  |  |  |  |  |
| **Cardiac disease** |  |  |  |  |  |  |  |
| Myocardial infarction | Past |  | 1.26(1.05 - 1.53) |  | 0.015 |  | 0.0019 |
|  | Recent |  | 1.37(1.12 - 1.67) |  | 0.002 |  |  |
| NYHA class | I & II |  | ref |  | - |  | <0.0001 |
|  | III & IV |  | 1.61(1.24 - 2.08) |  | <0.0001 |  |  |
| Heart failure |  |  | 1.54(1.32 - 1.80) |  | <0.0001 |  |  |
| Preoperative atrial fibrillation | |  | 1.43(1.09 - 1.88) |  | 0.009 |  |  |
| Diseased coronary vessels | None |  | ref |  | - |  |  |
|  | One |  | 1.35(1.04 - 1.76) |  | 0.027 |  |  |
|  | Two |  | 1.59(1.26 - 2.00) |  | <0.0001 |  |  |
|  | Three or more |  | 1.47(1.20 - 1.81) |  | 0.0002 |  |  |
| TEE Aortic stenosis |  |  |  |  |  |  |  |
| Less than moderate | |  | ref |  | - |  |  |
|  | Moderate |  | 1.26(0.88 - 1.79) |  | 0.20 |  |  |
| More than moderate | |  | 1.25(1.06 - 1.47) |  | 0.008 |  |  |
| TEE Aortic insufficiency |  |  |  |  |  |  |  |
| Less than moderate | |  | ref |  | - |  |  |
|  | Moderate |  | 0.83(0.67 - 1.03) |  | 0.97 |  |  |
| More than moderate | |  | 0.71(0.54 - 0.94) |  | 0.15 |  |  |
| Preoperative TTE Mitral regurgitation | |  |  |  |  |  |  |
| Less than moderate | |  | ref |  | - |  | 0.0008 |
|  | Moderate |  | 0.76(0.64 - 0.90) |  | 0.001 |  |  |
| More than moderate | |  | 0.64(0.48 - 0.84) |  | 0.001 |  |  |
| Intraoperative TEE Mitral regurgitation | |  |  |  |  |  |  |
| Less than moderate | |  | ref |  | - |  | <0.0001 |
|  | Moderate |  | 1.19(1.01 - 1.42) |  | 0.043 |  |  |
| More than moderate | |  | 1.67(1.36 - 2.05) |  | <0.0001 |  |  |
| Worst grade of Mitral regurgitation | |  |  |  |  |  |  |
|  | Moderate |  | ref |  | - |  |  |
| More than moderate | |  | 1.19(1.01 - 1.41) |  | 0.036 |  |  |
| LV ejection fraction <40% |  |  | 1.13(0.97 - 1.33) |  | 0.11 |  |  |
|  |  |  |  |  |  |  |  |
| **Operation** |  |  |  |  |  |  |  |
| Year of operation | 2002-2003 |  | ref |  |  |  |  |
|  | 2004-2005 |  | 0.94(0.75 - 1.17) |  | 0.56 |  |  |
|  | 2006-2007 |  | 0.87(0.69 - 1.10) |  | 0.25 |  |  |
|  | 2008-2009 |  | 0.74(0.57 - 0.95) |  | 0.020 |  |  |
|  | 2010-2011 |  | 0.75(0.57 - 0.98) |  | 0.037 |  |  |
|  | 2012-2013 |  | 0.66(0.47 - 0.92) |  | 0.014 |  |  |
|  | 2014-2015 |  | 0.54(0.28 - 1.03) |  | 0.062 |  |  |
| Urgency (Non-elective) |  |  | 1.51(1.30 - 1.75) |  | <0.0001 |  |  |
| CABG and/or AVR performed | AVR |  | ref |  | - |  | 0.0013 |
|  | CABG |  | 1.23(1.02 - 1.47) |  | 0.028 |  |  |
|  | CABG and AVR |  | 1.47(1.19 - 1.82) |  | 0.0004 |  |  |
| Mitral valve operation (All 1,515 patients) | |  | 1.00(0.86 - 1.17) |  | 1.00 |  |  |
| Mitral valve operation (Patients with moderate MR; N=1,112) | |  | 0.93(0.76 - 1.14) |  | 0.49 |  |  |
| Mitral valve operation (Patients with greater than moderate MR; N=403) | |  | 0.81(0.57 - 1.14) |  | 0.22 |  |  |
| Mitral valve repair or replacement | Repair |  | ref |  | - |  |  |
|  | Replacement |  | 1.12(0.83 - 1.51) |  | 0.45 |  |  |
|  |  |  |  |  |  |  |  |

**Supplemental Table 3: Cox-Snell proportional hazards residual model fit of the cohort** (N = 1515, number of events = 651; concordance=0.679; r2=0.172)

| **Variable** | **Level** | **Coefficient** | **Exp(coef)** | **SE(coef)** | **Z score** | **P value** |
| --- | --- | --- | --- | --- | --- | --- |
| Age | 50-59 | 0.51305 | 1.67038 | 0.33935 | 1.512 | 0.130564 |
|  | 60-69 | 1.01225 | 2.75179 | 0.31693 | 3.194 | 0.001403 |
|  | 70-79 | 1.47521 | 4.37196 | 0.30985 | 4.761 | 1.93E-06 |
|  | >=80 | 1.93374 | 6.91532 | 0.31201 | 6.198 | 5.73E-10 |
| Chronic lung disease | Yes | 0.51287 | 1.67007 | 0.09483 | 5.408 | 6.36E-08 |
| Dialysis | Yes | 1.48115 | 4.398 | 0.18393 | 8.053 | 7.77E-16 |
| Diabetes | IDDM | 0.34896 | 1.41759 | 0.12401 | 2.814 | 0.004893 |
|  | NIDDM | 0.22488 | 1.25217 | 0.0973 | 2.311 | 0.02083 |
| NYH Class | 3 | 0.27842 | 1.32104 | 0.08437 | 3.3 | 0.000966 |
|  | 4 | 0.13987 | 1.15012 | 0.13544 | 1.033 | 0.301762 |
| Hypercholesterolemia | Yes | -0.22224 | 0.80072 | 0.09051 | -2.455 | 0.014074 |
| Hypertension | Yes | 0.24161 | 1.2733 | 0.1019 | 2.371 | 0.017735 |
| Preoperative AF | Yes | 0.29137 | 1.33827 | 0.13975 | 2.085 | 0.037066 |
| Non-elective operation | Yes | 0.32771 | 1.38779 | 0.07988 | 4.102 | 4.09E-05 |

**Supplemental Figure 1. Plot of Cox-Snell proportional hazards residual model fit of the cohort.**


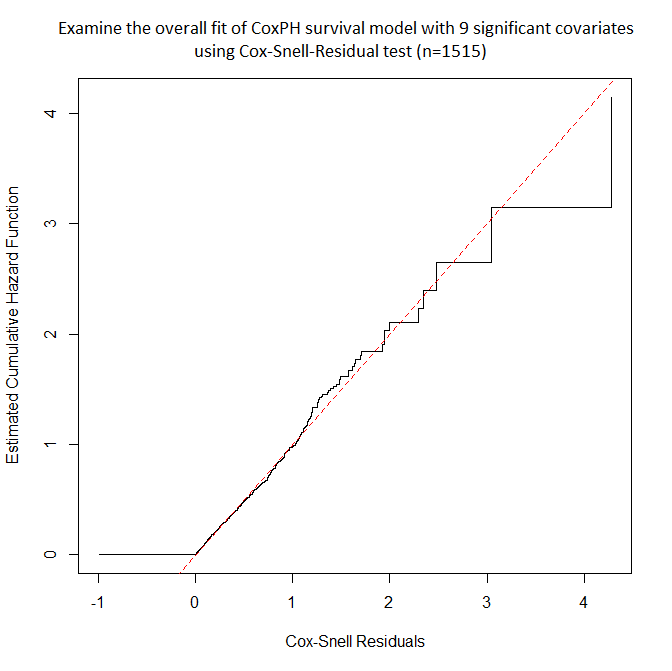

Supplement: Supplementary file 1 [file Datasheet1.docx]
